# Systematic literature review on the real-world effectiveness and safety of bempedoic acid

**DOI:** 10.1016/j.athplu.2026.100562

**Published:** 2026-03-24

**Authors:** Arrigo Francesco Giuseppe Cicero, Christopher P. Cannon, Raju Gautam, Saeed Anwar, Aishee Ghatak, Evelyn Sarnes, Heather A. Powell, Richa Chhabra, Shantanu Jawla

**Affiliations:** a Medical and Surgical Sciences Department, Alma Mater Studiorum University of Bologna, Bologna, Italy; b Cardiovascular Medicine Unit, IRCCS AOUBO, Bologna, Italy; c Division of Cardiovascular Medicine, Department of Medicine, Brigham and Women's Hospital, Harvard Medical School, Boston, MA, USA; d ConnectHEOR Limited, London, United Kingdom; e ConnectHEOR Pvt. Ltd., New Delhi, India; f Esperion Therapeutics, Inc., Ann Arbor, MI, USA; g Daiichi Sankyo Europe GmbH, Munich, Germany

**Keywords:** Bempedoic, Atherosclerosis, Hypercholesterolemia, Dyslipidemia, Statins, Cardiovascular

## Abstract

**Background and aims:**

Cardiovascular disease remains a significant global health burden. Bempedoic acid is a novel, oral adenosine triphosphate-citrate lyase inhibitor that lowers low-density lipoprotein cholesterol (LDL-C). While randomized controlled trials (RCTs) have established its efficacy and safety, evidence from real-world clinical practice is needed.

**Methods:**

A systematic literature review (SLR) was conducted to identify studies reporting real-world use of bempedoic acid as monotherapy, in fixed dose combination with ezetimibe, or in combination with any other lipid lowering therapy (LLT). MEDLINE, EMBASE, and Cochrane Library were searched from inception to February 19, 2025. Two reviewers independently screened studies, with discrepancies resolved by a third. Data extraction and validation were conducted, and the risk of bias assessed using the Newcastle–Ottawa Scale.

**Results:**

Of 269 records, 28 reports on 22 unique studies met the eligibility criteria. Most studies evaluated LDL-C changes from baseline after initiation of bempedoic acid; one pragmatic randomized trial was identified. Bempedoic acid achieved LDL-C reductions of 15% to 39% when added to maximally tolerated statins, 22% to 38% in statin intolerant cohorts, and 18% to 42% in mixed statin intolerant cohorts. Safety data was limited; treatment discontinuation ranged from 9% to 36%, with higher rates in cohorts with greater statin-intolerance.

**Discussion:**

This SLR shows bempedoic acid, with or without other LLTs, achieves a minimum of approximately 20% LDL-C reductions, consistent with RCTs. Data suggests it may be effective as an adjunct to statins and as an oral alternative for statin-intolerant patients.

## Highlights

• Real-world bempedoic acid achieved ≥20% LDL-C reduction in routine practice.
• LDL-C lowering was consistent across statin-treated and statin-intolerant cohorts.
• The magnitude of LDL-C reduction was broadly consistent with RCT evidence.
• No new safety concerns emerged, although safety reporting was limited.

## 1 Introduction

Despite the sustained reductions in cardiovascular disease (CVD) morbidity and mortality over the past four decades, CVD remains the leading cause of disease burden worldwide [1,2]. Approximately 17.8 million people die each year from CVD [1,3]. Elevated low-density lipoprotein cholesterol (LDL-C) is a well-established causal risk factor for major adverse cardiovascular events (MACE), including myocardial infarction and stroke [4,5]. To reduce cardiovascular risk, international guidelines have progressively lowered recommended LDL-C targets, with current thresholds set at <55 mg/dL (<1.4 mmol/L) for very high-risk individuals [4]. To achieve these targets, treatment typically requires lipid-lowering therapy (LLT), most commonly statins, which are supported by strong evidence for reducing cardiovascular events [4,6]. However, many patients fail to reach guideline-recommended LDL-C targets with statin therapy alone or are unable to tolerate statins because of adverse effects such as statin-associated muscle symptoms, leading to poor adherence [7].

Bempedoic acid is an oral lipid-lowering agent that inhibits adenosine triphosphate (ATP)-citrate lyase [8]. It is approved as an adjunct to diet in adults with primary hypercholesterolemia or mixed dyslipidemia, and in adults with established or at high risk for atherosclerotic cardiovascular disease (ASCVD) to reduce cardiovascular risk [9,10]. Like statins, bempedoic acid upregulates hepatic low-density lipoprotein (LDL) receptor expression, thereby reducing circulating LDL-C levels [11]. ATP-citrate lyase is positioned upstream of 3-hydroxy-3-methylglutaryl–coenzyme A (HMG-CoA) reductase in the cholesterol biosynthesis pathway. Unlike statins, bempedoic acid is a prodrug requiring activation by very-long-chain acyl-CoA synthetase-1 (ACSVL1), an enzyme expressed in the liver but not in skeletal muscle, which explains its lower incidence of muscle-related adverse effects [[12], [13], [14], [15]]. Recent mechanistic data also indicates that bempedoic acid does not impair mitochondrial respiratory function in skeletal muscle cells [16]. Bempedoic acid can be used in combination with a statin or other LLTs for patients who do not achieve LDL-C targets with statins alone, or with ezetimibe in those who are statin intolerant or have a contraindication to statins [9,10].

Clinical trials have shown bempedoic acid reduces LDL-C levels and the CLEAR Outcomes trial demonstrated a significant reduction in MACE compared with placebo in patients who are statin intolerant [[12], [13], [14], [15],^17^]. The drug has generally been well tolerated, although an increased risk of gout has been observed [18]. While randomized controlled trial (RCT) data have established the efficacy and safety of bempedoic acid, less is known about its real-world use. This systematic literature review (SLR) was conducted to assess the real-world effectiveness and safety of bempedoic acid in clinical practice, either as monotherapy or in combination with other LLTs.

## 2 Materials and methods

The SLR followed the guidance outlined in the Cochrane Handbook [19], and the reporting requirements of the Preferred Reporting Items for Systematic Reviews and Meta-Analyses (PRISMA) 2020 guidelines [20].

### 2.1 Literature search strategy

The literature searches were conducted via Ovid in MEDLINE, EMBASE, Cochrane Central Register of Controlled Trials (CENTRAL) and Cochrane Database of Systematic Reviews (CDSR), from database inception until February 19, 2025.

The search strategy combined free-text terms for hypercholesterolemia, dyslipidemia, CVD and LDL-C, and terms for bempedoic acid and real-world study designs. No limits were applied to ensure coverage of studies evaluating bempedoic acid. Search terms were derived based on a pre-existing SLR [21]. The full search strategy is provided in Supplementary Table 1.

Additional searches were conducted for abstracts presented at relevant scientific congresses hosted between 2022 and 2024 using terms such as “bempedoic”. Study authors also cross-checked their own reference lists to identify any publications not retrieved through the primary database searches. A full list of conferences searched is provided in Supplementary Table 2.

### 2.2 Eligibility criteria

Studies were eligible for inclusion if they met all of the following criteria:

1. Population: adults (≥18 years) with primary hypercholesterolemia or mixed dyslipidemia, and/or ASCVD, either established disease or at high risk;
2. Intervention: bempedoic acid monotherapy, bempedoic acid as a fixed-dose in combination with ezetimibe, or in combination with any other LLTs such as statins, ezetimibe, alirocumab, evolocumab, or inclisiran;
3. Comparator: any or no comparator;
4. Outcomes: LDL-C, high-sensitivity C-reactive protein (hsCRP), individual or composite cardiovascular events, all-cause mortality, treatment discontinuation or any adverse event (AE);
5. Study design: real-world observational studies (including pragmatic clinical trials); and
6. Language: published in English.

A full list of eligibility criteria, including population, intervention, comparator, outcomes (PICO), study design, and language is provided in Supplementary Table 3.

### 2.3 Selection of eligible studies

Study selection was conducted in two stages: at title and abstract, followed by full text, using a Microsoft® Excel based screening form. At each stage, two reviewers independently assessed studies for inclusion based on predefined eligibility criteria (SA and AG). Discrepancies were resolved by consensus, with a third reviewer consulted as needed (RG).

### 2.4 Data extraction

Data extraction was performed using a standardized data extraction template developed in Microsoft® Excel. Relevant information from the included studies was extracted by one reviewer (SA or AG) and independently validated by the other. Discrepancies were resolved by consensus, with a third reviewer consulted as needed (RG).

Key variables were extracted across three domains:

• Publication and study details: author, year, country, study design, data source, funding, sample size, and follow-up duration.
• Baseline characteristics: demographics (e.g., age, sex), comorbidities, baseline lipid levels (e.g., LDL-C), prior LLT use.
• Results: change from baseline in LDL-C levels; the proportion of patients who achieved LDL-C targets; change from baseline in hsCRP; proportion of patients who experienced a cardiovascular event; proportion of patients with all-cause mortality; treatment discontinuation rates; and safety outcomes, including the proportion of patients who experienced an AE, a serious adverse events (SAE), or specific AE.

### 2.5 Risk of bias assessment

Risk of bias was assessed using the Newcastle–Ottawa Scale (NOS), which evaluates observational studies across three domains: selection, comparability, and outcome (for cohort studies) or exposure (for case-control studies) [22].

Each study was independently assessed by one reviewer (SA or AG) and verified by the other. Discrepancies were resolved by consensus, with a third reviewer consulted as needed (RG).

For interpretation, NOS scores were categorized as follows:

• Low risk of bias: 7–9 points
• Moderate risk of bias: 5–6 points
• High risk of bias: <5 points

Studies assessed as having a high risk of bias were flagged during the qualitative synthesis of findings.

### 2.6 Data synthesis

A meta-analysis was not conducted due to substantial heterogeneity across the included studies in terms of design (prospective and retrospective), populations (e.g. statin intolerance, comorbidities, primary vs. secondary and mixed prevention), and inconsistencies in the definitions of statin intolerance. In addition, there was variability in the reporting of background LLT, outcome definitions (e.g. LDL-C targets) and reported timepoints. Instead, a narrative synthesis was conducted to describe the range and consistency of findings across studies.

Data on hsCRP, individual or composite cardiovascular events and all-cause mortality were limited, with fewer than three studies reporting on these outcomes. Given the restricted generalizability of such sparse evidence, these outcomes were excluded from the synthesis.

Results were summarized by outcome (LDL-C reduction, LDL-C targets and safety). For reporting LDL-C effectiveness, studies were further grouped in three categories:

• Bempedoic acid plus statins: studies where bempedoic acid was evaluated as add-on therapy to maximally tolerated statins, with or without other LLTs.
• Bempedoic acid in statin intolerant cohorts (or ≥80% statin intolerant): studies primarily involving patients unable to tolerate statins.
• Bempedoic acid in mixed statin intolerant cohorts or statin intolerance unknown: studies including heterogeneous populations with partial statin intolerance and diverse or unspecified LLT combinations.

For LDL-C reduction, the relevant effect measure was percentage change from baseline. For all other outcomes (LDL-C target and safety), we considered proportions of patients experiencing the outcome and/or the number of events reported. These effect measures were pre-specified based on their suitability for summarizing real-world observational data across both comparative and non-comparative study designs.

To ensure consistency across studies, timepoints reported in weeks were converted to months using the assumption that 1 month equals 4 weeks. LDL-C values reported in mmol/L were converted to mg/dL by multiplying by 38.67. Where LDL-C values were reported with decimals, these were rounded to the nearest whole number (mg/dL).

## 3 Results

### 3.1 Search results

**Fig. 1:**
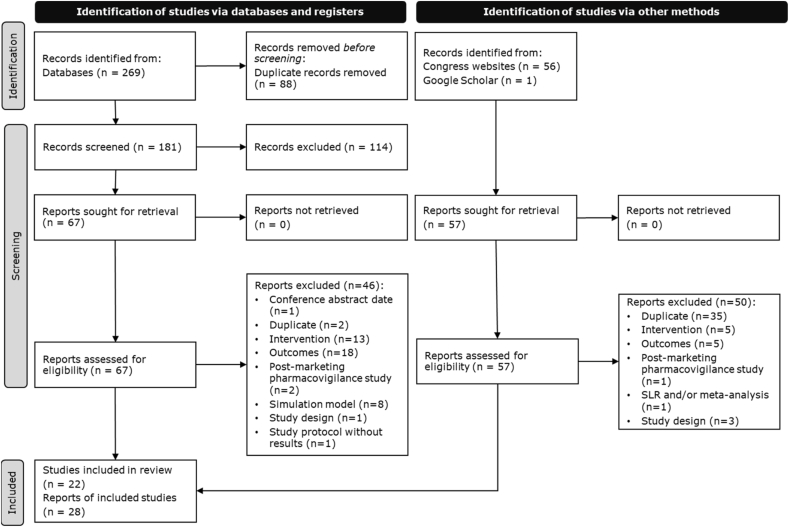
PRISMA flow of literature.

A total of 269 records were identified through database searching, of which 88 were duplicate records across bibliographic sources. After removing duplicate records, 181 unique records were screened. Of these, 67 reports were assessed for eligibility, and 46 were subsequently excluded for not meeting the inclusion criteria. The most common reasons for exclusion were lack of relevant outcomes or outcome data (n = 18) and irrelevant intervention (n = 13). An additional 57 records were identified from other sources, of which 7 met the eligibility criteria and were included. In total, 28 reports on 22 unique studies were included in the SLR [[23], [24], [25], [26], [27], [28], [29], [30], [31], [32], [33], [34], [35], [36], [37], [38], [39], [40], [41], [42], [43], [44]]. The PRISMA flow diagram is illustrated in Fig. 1.

### 3.2 Study and patient characteristics

This SLR identified real-world studies on the use of bempedoic acid worldwide, including in the United Kingdom (n = 6) [23,27,35,37,40,41], India (n = 4) [29,38,39,42], Italy (n = 5) [24,30,32,33,44], Germany (n = 3) [25,36,43], the United States (n = 2) [31,34] and one study each in Belgium [28] and Spain [26].

Twelve studies used prospective data collection [24,25,30,32,33,35,38,39,[41], [42], [43], [44]], and the remaining 10 studies were retrospective studies [23,[26], [27], [28], [29],^31^,^34^,^36^,^37^,^40^]. Thirteen studies were conducted within single-center institutions [[23], [24], [25], [26],^29^,^33^,^34^,[36], [37], [38], [39],^42^,^44^], five studies were in two or more centers [28,30,40,41,43]; the remaining studies did not report on number of study sites [27,31,32,35]. Sample sizes ranged from 15 to 1515 patients across studies.

Most real-world studies evaluated outcomes in relation to patients’ baseline values following initiation of bempedoic acid (with or without ezetimibe and other LLTs); with one open, pragmatic randomized trial with two parallel arms that assessed bempedoic acid versus statin titration [44].

The majority of studies were published as conference abstracts (n = 14) [24,[26], [27], [28], [29],[31], [32], [33], [34], [35], [36], [37], [38],^43^], with only a subset (n = 8) available as full-text peer-reviewed articles [23,25,30,[39], [40], [41], [42],^44^]. An overview of the included studies is presented in Table 1.

**Table 1:** Overview of the included studies.

Author year	Publication type	Country	Study design	Population	Classification	Primary or Secondary Prevention	Bempedoic acid regimen	Statin use/intolerant	Sample size
Berthold 2023 [43]	Abstract	Germany	Prospective	Primary hypercholesterolemia or mixed dyslipidemia	**Group 3**: Mixed statin intolerance	NR	BA ± ezetimibe	NR	992
Demeure 2023 [28]	Abstract	Belgium	Cross-sectional study	NR	**Group 3**: Mixed statin intolerance	**Mixed**; 47% had ASCVD	BA (not further specified)	65% statin intolerant	83
Donnarumma 2024 [24]	Abstract	Italy	Prospective	Hypercholesterolemia	**Group 2:** All patients statin-intolerant	**Primary**; no prior CV events	BA + ezetimibe	100% statin intolerant	19
Fazio 2024 [32]	Abstract	Italy	Prospective	ASCVD	**Group 1:** All patients on statins	**Secondary**; all patients had ASCVD	BA + rosuvastatin or atorvastatin + ezetimibe	All patients on statins	22
Gatto 2024 [33]	Abstract	Italy	Prospective (& retrospective)	High or very high CV risk; HIV infected	**Group 3**: Mixed statin intolerance	NR	BA (no further details)	75% received BA plus statin plus ezetimibe	32
Jaiswal 2024 [38]	Abstract	India	Prospective	ASCVD	**Group 1:** All patients on statins	**Secondary**; all patients had ASCVD	BA + rosuvastatin + ezetimibe	All patients on statins	104
Jakubowska 2024 [41]	Full text	United Kingdom	Prospective	Mixed population	**Group 2:** All patients statin-intolerant (≥80%^a^)	**Mixed**; 19% had ASCVD	BA ± LLT	92% statin intolerant	213
Jariwala 2023 [29]	Abstract	India	Retrospective	>3 risk factors without a history of CVD	**Group 2:** All patients statin-intolerant	**Primary**; no history of CVD	BA monotherapy	100% statin intolerant	65
Joy 2023 [34]	Abstract	United States	Retrospective	Mixed population; ASCVD, FH, hyperlipidemia	**Group 2:** All patients statin-intolerant	**Mixed**; 35% had ASCVD (25% among patients who received bempedoic acid)	BA (no further details)	100% statin intolerant at baseline	35
Mahajan 2024 [42]	Full text	India	Prospective	ASCVD	**Group 1:** All patients on statins	**Secondary**; all patients had ASCVD	BA + rosuvastatin + ezetimibe	All patients on statins; initially statin naïve	128
Makhmudova 2023 [25]	Full text	Germany	Prospective	ASCVD	**Group 1:** All patients on statins	**Secondary**; all patients had ASCVD	BA + atorvastatin + ezetimibe	All patients on statins	85
Marazzi 2024 [44]	Full text	Italy	Prospective	Hypercholesterolemia	**Group 1:** All patients on statins	**Primary**; no prior CVD	BA + statin + ezetimibe vs statin titration	All patients on statins	120
Mccarron 2022 [35]	Abstract	United Kingdom	Prospective (& retrospective)	High CV risk	**Group 2:** All patients statin-intolerant	**Mixed**; 93% treated for primary prevention	BA ± ezetimibe	100% statin intolerant	15
Moghadam 2024 [37]	Abstract	United Kingdom	Retrospective	Mixed	**Group 2:** All patients statin-intolerant (≥80%^a^)	**Mixed**; 22% had prior ASCVD	BA ± ezetimibe	86% statin intolerance	100
Nelson 2024 [31]	Abstract	United States	Retrospective	Primary hyperlipidemia	**Group 3**: Mixed statin intolerance	**Mixed**; all patients had high risk for, or with, CV disease	BA ± ezetimibe	Most patients had evidence of statin use in the prior 12-months (58% to 65%)	1515
Ramachandran 2024 [40]	Full text	United Kingdom	Retrospective	Primary hypercholesterolemia or mixed dyslipidemia	**Group 2:** All patients statin-intolerant (≥80%^a^)	**Mixed**; 17% had prior CVD and 6% had a history of stroke and/or transient ischemic attack	BA ± LLT	93% statin intolerant	221
Rana 2025 [39]	Full text	India	Prospective	ASCVD	**Group 1:** All patients on statins	**Secondary**; all patients had ASCVD	BA + statins	All patients on statins	200
Russo 2025 [30]	Full text	Italy	Prospective	Dyslipidemia	**Group 3**: Mixed statin intolerance; subgroup data on **Group 1**: all patients on statins.	**Mixed**; 67% had chronic coronary syndrome, 9% had a recent ACS, 23% PAD, 2% stroke/TIA	Subgroup(s): BA + ezetimibe; BA + rosuvastatin + ezetimibe; BA + atorvastatin + ezetimibe	Mixed: 70.3% were on statins; subgroup data reported for patients with and without statin therapy	111
Schumann 2022 [36]	Abstract	Germany	Retrospective	Lipid clinic outpatients	**Group 2:** All patients statin-intolerant (≥80%^a^)	**NR**	Subgroup(s): BA + PCSK9i	80% statin intolerant	NR
Toledo 2024 [26]	Abstract	Spain	Retrospective	Heterozygous familial hypercholesterolemia and/or established vascular disease	**Group 3**: Mixed statin intolerance	**Mixed**; 50% had heterozygous familial hypercholesterolemia, 50% had an established vascular disease; 8.3% had both	BA ± LLT	Mixed; 54% on statins	24
Warden 2022 [23]	Full text	United Kingdom	Retrospective	Mixed; 89% ASCVD, 100% hyperlipidemia, 64% FH	**Group 3**: Mixed statin intolerance	**Mixed**; 89% had ASCVD	BA ± LLT	Mixed; 74% statin intolerant	73
Win 2024 [27]	Abstract	United Kingdom	Retrospective	Mixed	**Group 3**: Mixed statin intolerance	**Mixed**; 49% were treated for primary prevention, 51% for secondary prevention	BA ± LLT	NR (all patients were previously on statins)	37

Abbreviations: ASCVD, Atherosclerotic cardiovascular disease; BA, bempedoic acid; CVD, cardiovascular disease; FH, familial hypercholesterolemia; LLT, lipid-lowering therapy; NR, not reported; PCSK9i, PCSK9 inhibitor.

a If ≥ 80% of the study cohort was reported as statin-intolerant, the study was grouped with those in which all patients were statin-intolerant. This threshold was applied to ensure consistent classification of predominantly statin-intolerant populations and to minimize misclassification when statin tolerance status was incompletely reported or represented a smaller proportion of the cohort.

Among the eight full-text publications, seven studies were considered moderate quality [23,25,[39], [40], [41], [42],^44^] and one as low quality [30], as assessed via the NOS. The results of the risk of bias assessment are presented in Supplementary Table 4.

Where reported, the mean or median ages ranged from 54 to 68 years. At baseline, prior to initiation of bempedoic acid, patients had LDL-C levels ranging from 90 mg/dL to 146 mg/dL.

Diabetes was present in 11% to 66% of patients [23,25,29,30,32,38,39,41,42]; hypertension in 33% to 83% [23,25,29,30,32,39,42,44]; and one study focusing on HIV reported HIV in 100% of patients [28].

Eleven studies evaluated bempedoic acid use in mixed primary and secondary prevention populations [23,[26], [27], [28],^30^,^31^,^34^,^35^,^37^,^40^,^41^]; five studies reported on secondary prevention only [25,32,38,39,42]; three on primary prevention only [24,29,44]; and three did not clearly report cardiovascular risk status [33,36,43]. Further disease characteristics are reported in Table 1.

### 3.3 Treatment characteristics

Studies were classified according to the reported bempedoic acid treatment regimen, including statin use and statin intolerance status, and were grouped as follows; of note, one study had data that contributed to two groups [30]:

• Bempedoic acid added to maximally tolerated statins (n = 7) [25,30,32,38,39,42,44];
• Bempedoic acid in patients who were statin-intolerant (≥80% statin intolerant) (n = 8) [24,29,[34], [35], [36], [37],^40^,^41^]; and
• Bempedoic acid in a mixed cohorts with statin intolerance or statin intolerance unknown (n = 8) [23,[26], [27], [28],^30^,^31^,^33^,^43^].

### 3.4 LDL-C reduction

#### 3.4.1 Bempedoic acid plus statins

In the only pragmatic clinical trial identified in the SLR, primary prevention patients with hypercholesterolemia already on high-intensity statins and ezetimibe for at least 3 months, were randomized to receive bempedoic acid (in addition to statins and ezetimibe), or statin dose titration. At 3 months, bempedoic acid achieved a 22.9% LDL-C reduction (baseline LDL-C = 90 mg/dL), compared with an 8% reduction with statin titration alone (baseline LDL-C = 86 mg/dL; between group comparison: p = 0.002) [44].

In a prospective study in secondary prevention patients with coronary artery disease (CAD) on stable, moderate-to high-intensity statin therapy, escalation to bempedoic acid lowered LDL-C by 15.2% after 3 months (baseline LDL-C = 120 mg/dL) and 21.5% after 12 months compared with baseline (p < 0.001) [39].

In secondary prevention patients with ASCVD, a small subgroup of patients (n = 8) already on high-intensity atorvastatin plus ezetimibe achieved a 15.0% LDL-C reduction at 1 month after escalation to bempedoic acid (baseline LDL-C = 124 mg/dL) [25]. Further reductions at 6 months were observed in 2 studies in patients with ASCVD on maximum dose of rosuvastatin plus ezetimibe [38], or rosuvastatin/atorvastatin plus ezetimibe (intensity not reported) [32]. In these studies, the initiation of bempedoic acid achieved LDL-C reductions of 29.1% (baseline LDL-C = 100 mg/dL) to 39.0% (baseline LDL-C = 130 mg/dL) at 6 months’ follow-up [32,38].

In a prospective study of secondary prevention following recent acute coronary syndrome (ACS) in patients who were statin-naïve, more substantial LDL-C reductions of 61.9% were achieved within 1-month of initial triple therapy with bempedoic acid plus high-intensity rosuvastatin and ezetimibe (baseline LDL-C = 116 mg/dL) [42].

In a prospective study on patients with dyslipidemia and mixed prevention status, the initiation of bempedoic acid to ongoing high-intensity atorvastatin and ezetimibe achieved a 26.3% LDL-C reduction at 2 months (baseline LDL-C = 78 mg/dL) [30]. In the same study, LDL-C reductions were in a similar range (baseline LDL-C = 78 mg/dL; 29.8% reduction) at 2 months after initiation of bempedoic acid to high intensity rosuvastatin and ezetimibe [30].

**Fig. 2:**
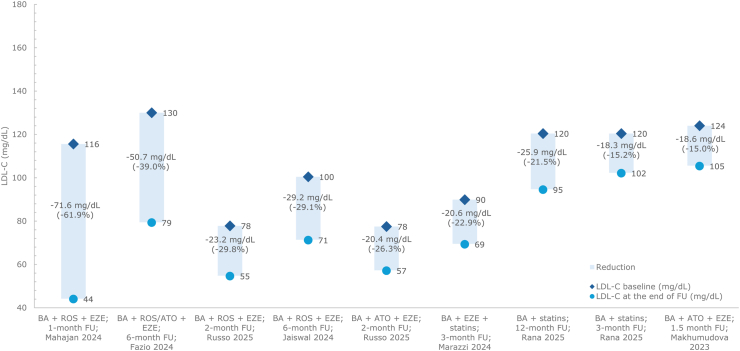
LDL-C reduction from baseline: bempedoic acid plus statins Abbreviations: ATO, atorvastatin; BA, bempedoic acid; EZE, ezetimibe; FU, follow-up; LDL-C, low-density lipoprotein cholesterol; ROS, rosuvastatin.

Data on LDL-C reduction for bempedoic acid plus statins are presented in Fig. 2.

#### 3.4.2 Bempedoic acid in statin-intolerant cohorts

In a primary prevention cohort (n = 65) without ASCVD but with >3 cardiovascular risk factors and contraindications to statin therapy (baseline LDL-C = 139 mg/dL), bempedoic acid monotherapy achieved a 25.6% LDL-C reduction at 9-month follow-up [29].

In a small, primary prevention study (n = 19) of statin intolerant patients with hypercholesterolemia and no prior cardiovascular events, bempedoic acid added to ongoing, maximally tolerated LLT reduced LDL-C by 38.5% at 1-month follow-up (baseline LDL-C = 123 mg/dL) [24]. All patients were on ezetimibe and 16% were on PCSK9i [24].

A small prospective study (n = 15) in high cardiovascular risk patients with statin intolerance, most of whom were treated for primary prevention (n = 14), evaluated bempedoic acid given as monotherapy (n = 3) or in combination with ezetimibe (n = 12) [35]. After 3 months, only 8 patients remained on bempedoic acid; these patients achieved a 32.3% LDL-C reduction (baseline LDL-C = 179.0 mg/dL) [35]. A retrospective analysis was conducted on electronic health records in a mixed prevention cohort that received bempedoic acid as monotherapy or in the fixed combination with ezetimibe (further details on background LLT not specified); the majority of patients were statin intolerant (86%) [37]. At 12 months, mean LDL-C was reduced by 25.9% in the overall population (baseline LDL-C = 124 mg/dL) and by 23.3% in the secondary prevention subgroup (baseline LDL-C = 114 mg/dL) compared to baseline [37].

Similar LDL-C reductions were reported in three additional studies in predominantly statin-intolerant patients on a variety of background LLTs [40,41], or unclear reporting of background LLTs [34]. In patients with primary hypercholesterolemia or mixed dyslipidemia, most of whom were statin intolerant (93%), bempedoic acid, initiated in line with the Summary of Product Characteristics, produced a 22% LDL-C reduction at 3 months (baseline LDL-C = 155 mg/dL); over half (54%) of patients continued statins, the majority on low- (74%) or medium-intensity statins (13%), while others received background LLTs including ezetimibe [40]. In a mixed prevention cohort that included patients with monogenic familial hypercholesterolemia (31%), drug intolerance to at least two statins was reported in 89% of patients; 15% continued some statin therapy (e.g., intermittent dosing) and 42% received ezetimibe. In this study, bempedoic acid achieved a 27% LDL-C reduction at 22-month follow-up (baseline LDL-C = 164 mg/dL) [41]. Lastly, in a small mixed-prevention cohort, four patients received bempedoic acid and achieved a 34% LDL-C reduction at 3 months (baseline LDL-C = 146 mg/dL); no further treatment details were provided [34].

**Fig. 3:**
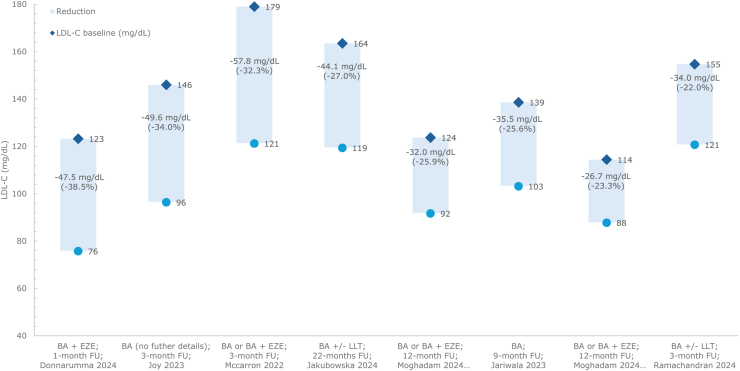
LDL-C reduction from baseline: bempedoic acid in statin intolerant patients Abbreviations: BA, bempedoic acid; EZE, ezetimibe; LLT, lipid-lowering therapy; FU, follow-up; LDL-C, low-density lipoprotein cholesterol.

Data on LDL-C reduction with bempedoic acid in statin-intolerant cohorts (≥80% statin intolerant) are presented in Fig. 3.

#### 3.4.3 Bempedoic acid in mixed cohorts with unknown statin intolerance status

In a large retrospective analysis of patients with a prescription claim for bempedoic acid, regardless of cardiovascular risk status, bempedoic acid monotherapy reduced LDL-C by 18.0% at 3 months (baseline LDL-C = 137 mg/dL), with reductions sustained at 12 months versus baseline (p < 0.0001) [31]. Patients on bempedoic acid plus ezetimibe achieved a 28.0% reduction at 3 months, with reductions of 22% sustained at 12 months versus baseline (baseline LDL-C = 131 mg/dL) (p < 0.001) [31].

In the MILOS study, a large European prospective, observational study in adults with primary hypercholesterolemia or mixed dyslipidemia (n = 992) who were treated with bempedoic acid or the fixed-dose combination with ezetimibe (baseline LDL-C = 124 mg/dL), results at 1 year indicated a mean reduction of 27.3% and at 2 years, a reduction of 30.3% [43]. In a small subgroup of patients (n = 18) from a prospective study on patients with dyslipidemia and mixed prevention status, patients on ongoing ezetimibe who initiated bempedoic acid achieved an LDL-C reduction of 42.0% at 2 months (baseline LDL-C = 84 mg/dL) [30].

Four additional real-world studies reported LDL-C reductions in the range of 25% to 39% from baseline LDL-C values of 98 to 141 mg/dL, although details on whether bempedoic acid was used as monotherapy or in fixed combination with ezetimibe were not consistently specified [23,26,28,33]. One study in a mixed prevention cohort reported that 73% of patients on bempedoic acid had an LDL-C reduction of greater than 21%; as this was a conference abstract only, no further details were provided [27].

**Fig. 4:**
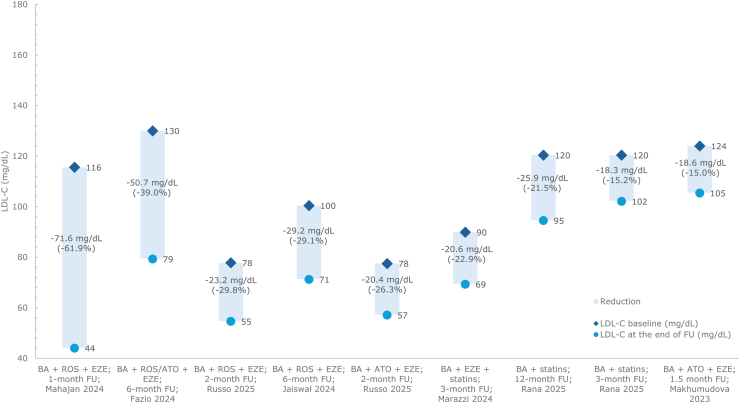
LDL-C reduction from baseline: bempedoic acid in mixed cohorts with statin intolerance Abbreviations: BA, bempedoic acid; FU, follow-up; LDL-C, low-density lipoprotein cholesterol; LLT, lipid-lowering therapy.

Data on LDL-C reduction for bempedoic acid in a mixed statin intolerant cohort and background LLTs are presented in Fig. 4.

### 3.5 LDL-C targets

#### 3.5.1 Bempedoic acid plus statins

When used as primary prevention in patients with hypercholesterolemia receiving high-intensity statins plus ezetimibe, 63% of those randomized to bempedoic acid achieved an LDL-C target of <70 mg/dL at 3 months (baseline LDL-C = 89.9 mg/dL), compared with 37% of patients randomized to statin titration alone (baseline LDL-C = 87.5 mg/dL). The difference between groups was statistically significant (p = 0.034) [44].

In statin-naïve patients with acute ASCVD following recent ACS, from a baseline value of 115.6 mg/dL, 71.9% achieved LDL-C targets of <50 mg/dL (based on Lipid Association of India [LAI] guidelines) within 1 month of initiating triple therapy with bempedoic acid plus high intensity rosuvastatin and ezetimibe [42]. When setting the LDL-C targets at <70 mg/dL, 92.6% of patients with acute ACS on triple therapy achieved this target [42].

In secondary prevention patients with ASCVD, among a small subgroup of patients (n = 8) already on high-intensity atorvastatin plus ezetimibe, all patients escalated to bempedoic acid achieved an LDL-C target of <54 mg/dL within 1.5 months of follow-up (baseline LDL-C = 124 mg/dL) [25].

**Fig. 5:**
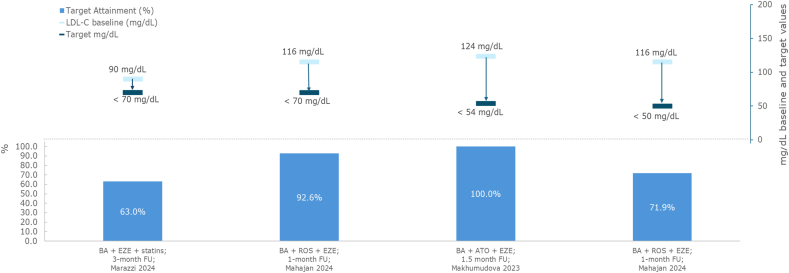
Target LDL-C (mg/dL) attainment: bempedoic acid plus statins Abbreviations: ATO, atorvastatin; BA, bempedoic acid; EZE, ezetimibe; FU, follow-up; LDL-C, low-density lipoprotein cholesterol; ROS, rosuvastatin.

Data on the attainment of LDL-C targets for bempedoic acid plus statins are presented in Fig. 5.

#### 3.5.2 Bempedoic acid in statin-intolerant cohorts

In a small, primary prevention study (n = 19), 32.0% of patients with hypercholesterolemia and statin intolerance already on maximally tolerated LLT (all patients were on ezetimibe and 16.0% on PCSK9i), achieved an LDL-C target <70 mg/dL (baseline value = 123 mg/dL), and 90.0% reached <100 mg/dL at 1 month after initiating bempedoic acid [24].

In another small subgroup of patients with ASCVD (n = 20), no patients who received bempedoic acid as monotherapy or the fixed combination with ezetimibe (further details on background LLT not specified) achieved an LDL-C target of <54 mg/dL after 1 year. As the baseline LDL-C was 114 mg/dL, achieving this lower LDL-C target would have required an approximately 53.0% LDL-C reduction from baseline [37].

Two studies reported on the achievement of LDL-targets in predominantly statin-intolerant patients on a variety of background LLTs [40,41]. In one study in a mixed prevention cohort that included patients with monogenic familial hypercholesterolemia (31%), 40% of patients on bempedoic acid achieved a target of <97 mg/dL, and 20.0% achieved this when the target was set at <77 mg/dL (baseline LDL-C = 164 mg/dL) [41]. A second study in patients with primary hypercholesterolemia or mixed dyslipidemia, most of whom were statin intolerant (93%) found that 58.0% on bempedoic acid, initiated in line with the Summary of Product Characteristics achieved a target of <70 mg/dL (baseline LDL-C = 155 mg/dL) [40].

**Fig. 6:**
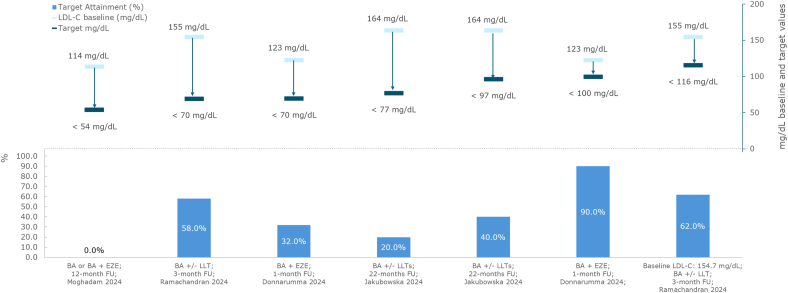
Target LDL-C (mg/dL): bempedoic acid in statin-intolerant patients Abbreviations: BA, bempedoic acid; EZE, ezetimibe; FU, follow-up; LDL-C, low-density lipoprotein cholesterol.

Data on the attainment of LDL-C targets with bempedoic acid in statin-intolerant populations (≥80% statin intolerant) are presented in Fig. 6.

#### 3.5.3 Bempedoic acid in mixed cohorts with unknown statin intolerance status

In a large retrospective analysis of patients with a prescription claim for bempedoic acid, regardless of cardiovascular risk status, 42.0% treated with bempedoic acid monotherapy (baseline LDL-C = 137 mg/dL) and 67.0% with bempedoic acid plus ezetimibe (baseline LDL-C = 131 mg/dL) achieved an LDL-C target of <100 mg/dL [31].

Across two real-world studies involving patients with varied statin tolerability, the proportion achieving LDL-C targets on bempedoic acid, ranged from 12.0% reaching <55 mg/dL [33] to 20.5% achieving <70 mg/dL (baseline LDL-C = 120 mg/dL) [23] after 3 [33], and 12 months of treatment [23].

**Fig. 7:**
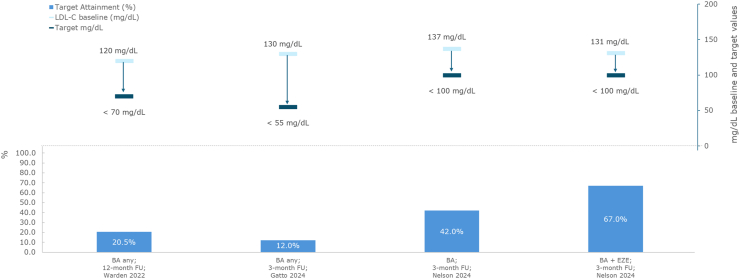
Target LDL-C (mg/dL): bempedoic acid in mixed cohorts with statin intolerance Abbreviations: BA, bempedoic acid; FU, follow-up; LDL-C, low-density lipoprotein cholesterol; LLT, lipid-lowering therapy.

Data on the attainment of LDL-C targets for bempedoic acid in mixed statin intolerant patients and background LLTs are presented in Fig. 7.

### 3.6 Safety

**Fig. 8:**
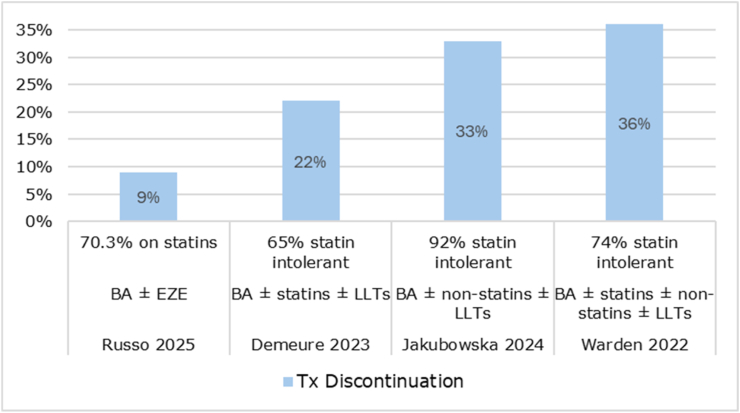
Safety outcomes: treatment discontinuation with bempedoic acid Abbreviations: BA, bempedoic acid; EZE, ezetimibe; LLT, lipid-lowering therapy; Tx, treatment.

Real-world data on the safety profile of bempedoic acid were limited. These findings should be interpreted with caution given the small number of studies. Among the few studies reporting treatment discontinuation (n = 4) [23,28,30,40], rates ranged from 9% to 36% across heterogeneous populations with mixed prevention status, varying degrees of statin intolerance, and different background LLTs. Higher discontinuation rates were observed in studies with a greater proportion of statin-intolerant patients (see Fig. 8). One study found that patients who discontinued bempedoic acid had a higher number of previously non-tolerated statins (2.86 vs 2.41; p = 0.02) and a greater number of total lipid-lowering drug classes not tolerated (1.89 vs 1.46; p = 0.003) compared to those who remained on treatment [41].

Across studies that reported specific AEs, bempedoic acid was generally well tolerated, with myalgia reported in 6% of patients (12 of 200) [39]; gout in approximately 2% to 3% of patients (2 of 83 to 2 of 65) [28,29]; elevated liver enzymes in 1.2% to 3% of patients (1 of 85 to 6 of 200) [28,39]; cholelithiasis in 2.7% of patients (2 of 65) [29]; and new-onset diabetes in 2% of patients (4 of 200) [39].

Where reported, the incidence of SAEs was low [39,43]. Fewer than 1% of patients (6 of 714) receiving bempedoic acid with or without ezetimibe experienced a SAE [43]. In a separate study, 4% of patients (8 of 200) on bempedoic acid plus statin therapy experienced a SAE, although SAEs were primarily due to underlying CAD rather than the study drug [39].

## 4 Discussion

The SLR suggests that in real-world practice, bempedoic acid, with or without other LLTs, achieves a minimum of approximately 20% LDL-C reductions, consistent with the magnitude of effect demonstrated in RCTs. These reductions were reported across both primary (23% to 39%) [24,29,44] and secondary prevention populations (15% to 39%) [25,32,38,39]. Consistent effects were also seen when bempedoic acid was added to statins (15% to 39%) [25,30,32,38,39,44], and when used in statin intolerant patients (22% to 38%) [24,29,34,35,37,40,41], despite the latter group having higher baseline LDL-C. In all-comer populations with mixed statin-intolerance and varied background LLTs, LDL-C reductions also ranged from 18% to 42% [23,26,28,30,31,33,43].

Safety findings in the real-world setting were generally aligned with those reported in RCTs and meta-analyses [[12], [13], [14], [15],^17^,^18^], although reporting was limited and inconsistently captured. An increased incidence of gout was observed with bempedoic acid [28,29]; higher discontinuation rates observed (up to 36%) [23,28,30,40] likely reflect the broader inclusion of patients with statin intolerance or LLT non-tolerance, although reasons for discontinuation were not consistently reported. No new safety concerns were reported. Overall, these findings are consistent with the established safety profile of bempedoic acid and support its use within stepwise treatment strategies, including as an adjunct to statins (with or without ezetimibe), but also as an oral alternative in patients unable to tolerate statins.

The 2025 ESC/EAS guidelines for the management of dyslipidemia provide new recommendations for bempedoic acid in both patients unable to take statins and as an adjunct to maximally tolerated statin therapy, with or without ezetimibe, including use in high- and very high-risk patients [45]. The recommendations position bempedoic acid alongside other established LLTs with demonstrated cardiovascular-risk reduction such as statins, ezetimibe, and PCSK9i. Importantly, the guidelines highlight that variability in LDL-C lowering is observed across all treatment options, likely reflecting differences in background LLT regimens [45]. In line with this, they emphasize the importance of monitoring LDL-C response 4 to 6 weeks after initiation or intensification and making timely treatment adjustments to achieve recommended targets [45].

Guidelines also recommend that the choice of treatment should not only be made based on the magnitude of additional LDL-C lowering needed, but also patient preference, treatment availability, and cost [45]. A recent discrete choice experiment found that patients expressed a strong preference for daily oral non-statin LLT, with a willingness-to-pay of $131 to $175 per month for an oral option that achieved an approximate 25% reduction in LDL-C levels [46]. Together, the growing body of evidence supports the use of bempedoic acid in clinical practice, both as adjunct to statins and as an oral alternative for patients with statin intolerance. Its consistent LDL-C–lowering effects and tolerability profile as observed in this real-world SLR, and favorable oral route of administration make it a practical and accessible option within lipid management pathways. Further, bempedoic acid has also been shown to provide improved lifetime cardiovascular risk reduction compared with standard of care at common cost-effectiveness thresholds ($150,000 per QALY), with additional value when combined with ezetimibe ($40,000 per QALY), and in those with statin-intolerance or those with heterozygous familial hypercholesterolemia [47].

Despite clear guidance, clinical inertia remains a barrier to LDL-C target attainment in routine practice. Recent data suggest that oral agents such as bempedoic acid, with demonstrated LDL-C–lowering efficacy and cardiovascular outcomes data, may facilitate treatment intensification in patients who remain above recommended targets [48]. However, implementation of guideline-directed lipid management remains complex in routine care, and real-world data continue to demonstrate that many high- and very high-risk patients do not achieve recommended LDL-C goals despite increased use of combination therapy [49]. In this context, recent debate contrasting “fire-and-forget” with personalized, goal-directed lipid strategies highlights that passive approaches may contribute to persistent undertreatment, whereas structured monitoring and predefined escalation pathways may improve LDL-C goal attainment [50]. Within such pragmatic care models, incorporation of additional oral non-statin therapies may support more timely and acceptable treatment intensification [50].

This SLR has limitations. The evidence-base was heterogeneous with respect to study design (e.g., prospective vs retrospective), patient populations (ASCVD, primary, and mixed prevention cohorts), baseline LDL-C levels and cardiovascular risk profiles. Substantial variability was also observed in the definition and degree of statin intolerance and in the reporting of background LLTs, including statin intensity, duration of prior therapy, and concomitant use of ezetimibe or PCSK9 inhibitors. As bempedoic acid is a recently licensed treatment, much of the real-world evidence is available only as conference abstracts, with a minority published as full-text, peer-reviewed articles, restricting access to detailed data. Additionally, most included studies evaluated outcomes relative to patients’ baseline values following initiation of bempedoic acid, and only one open, pragmatic randomized trial with parallel arms was identified, which limits the ability to draw conclusions regarding comparative effectiveness between treatment options. Reporting of cardiovascular outcomes and mortality was often incomplete, and tolerability data were inconsistently captured, limiting assessment of longer-term clinical implications. Lastly, LDL-C targets varied across studies, reflecting differences in guidelines and local recommendations, which limits direct cross-study comparisons of goal attainment. These limitations, together with the potential for publication and reporting bias, should be considered when interpreting the findings. An updated SLR is warranted as additional studies have been published since the predefined search cut-off date [[51], [52], [53]]. As further full-text publications become available, an updated review may enable more robust synthesis, including subgroup analyses and potential meta-analysis.

## 5 Conclusions

This SLR suggests that the addition of bempedoic acid, with or without other LLTs, achieves a minimum of approximately 20% LDL-C reductions, consistent with those observed in RCTs. It can be used both as an adjunct to statins and as an oral option for patients with statin intolerance. These findings support confidence in its effectiveness and tolerability in routine clinical practice. Larger, well-reported observational studies are still needed to confirm long-term cardiovascular outcomes in statin tolerant patients.

## Funding

This work was supported by Daiichi Sankyo Europe GmbH and conducted by ConnectHEOR Ltd. GIPAM GmbH was paid for medical writing support. (No grant ID is available)

## Declaration of competing interest

The authors declare the following financial interests/personal relationships which may be considered as potential competing interests:Arrigo Francesco Giuseppe is a scientific consultant for Zentiva SA, Dompé SpA and Italfarmaco SpA.Christopher P. Cannon reports in calendar years 2022 to 2024 – Research Grants from: Amgen, Better Therapeutics, Boehringer-Ingelheim (BI), Novo Nordisk, and salary support from Colorado Prevention Center (CPC) Clinical Research, which gets research grant support from Amgen, Bayer, Cleerly, Esperion, Lexicon, Silence; Consulting fees from: Amryt/Chiesi, Amgen, Ascendia, Biogen, BI, BMS, CSL Behring, Genomadix, Lilly, Janssen, Lexicon, Milestone, Novartis, Pfizer, Rhoshan. They serve on Data and Safety Monitoring Boards for the Areteia, Novo Nordisk, ROMTherapy, Inc. and the Veterans Administration.Shantanu Jawla and Richa Chhabra are employees of Daiichi Sankyo Europe GmbH (Study Sponsor).Evelyn Sarnes and Heather A. Powell are employees of ESPERION Therapeutics, Inc.Raju Gautam, Saeed Anwar, and Aishee Ghatak are employees of ConnectHEOR and received consulting fees for the conduct of this study.
