# Supplementary material for: Systematic literature review on the real-world effectiveness and safety of bempedoic acid
Source: Atheroscler Plus. 2026 Mar 24;65:100562. doi: 10.1016/j.athplu.2026.100562 (PMC13101281; doi:10.1016/j.athplu.2026.100562)
Supplement: Multimedia component 1 [file mmc1.docx]

Supplementary Table 1. Search Strategy for EMBASE, MEDLINE, CENTRAL and CDSR (via Ovid.com 19 February 2025)

| # | Query | Hits |
| --- | --- | --- |
| 1 | hypercholesterolemia.mp. | 155,481 |
| 2 | mixed dyslipidemia.mp. | 1,370 |
| 3 | heterozygous familial hypercholesterolemia.mp. | 3,055 |
| 4 | atherosclerosis.mp. | 481,018 |
| 5 | cardiovascular disease.mp. | 747,624 |
| 6 | cardiovascular.mp. | 2,236,152 |
| 7 | (Low density lipoprotein or LDL-C or LDL C).mp. | 415,136 |
| 8 | (coronary artery disease or peripheral artery disease or atherosclerotic cardiovascular disease or acute coronary syndrome or myocardial infarction or stroke or angina or ACS or PAD or ACD or primary prevention or secondary prevention).mp. | 2,475,111 |
| 9 | 1 or 2 or 3 or 4 or 5 or 6 or 7 or 8 | 4,662,962 |
| 10 | bempedoic acid.mp. | 1,803 |
| 11 | (bempedoic acid or nilemdo or nustendi or etc-1002 or etc1002 or nexletol or nexlizet).mp. | 1,857 |
| 12 | 10 or 11 | 1,857 |
| 13 | 9 and 12 | 1,687 |
| 14 | real world.mp. | 294,893 |
| 15 | real-world evidence.mp. | 20,396 |
| 16 | real world data.mp. | 45,024 |
| 17 | real world analysis.mp. | 3,684 |
| 18 | (real-world or real world or real world evidence or real-world evidence or electronic medical record or longitudinal or cohort or claims analysis or claim or insurance analysis or insurance or prospective or retrospective or real life or real-life or case control or case-control or registry or registries or cross-sectional or registr* or health survey or patient survey or physician survey or crosssectional).mp. | 11,837,748 |
| 19 | prospective.mp. | 2,865,330 |
| 20 | retrospective.mp. | 3,679,912 |
| 21 | claims analysis.mp. | 2,330 |
| 22 | (chart review or audit or case series or case-series).mp. | 639,196 |
| 23 | 14 or 15 or 16 or 17 or 18 or 19 or 20 or 21 or 22 | 12,089,113 |
| 24 | 13 and 23 | 269 |

Supplementary Table 2. Conference Search

| European Society of Cardiology (ESC): <https://www.escardio.org/> |
| --- |
| ESC Preventive Cardiology: <https://www.escardio.org/Congresses-Events/Preventive-Cardiology> |
| European Atherosclerosis Society (EAS): <https://eas-society.org/> |
| European Association for the Study of Diabetes: <https://www.easd.org/> |
| German Society of Cardiology (DGK): <https://dgk.org/> |
| American Heart Association (AHA): <https://www.heart.org/> |
| American College of Cardiology (ACC): <https://www.acc.org/> |
| National Lipid Association (NLA): <https://www.lipid.org/> |
| American Society of Preventive Cardiology (ASPC): <https://www.aspconline.org/> |
| Academy of Managed Care Pharmacy (AMCP): <https://www.amcp.org/> |

Supplementary Table 3. SLR Eligibility Criteria

| Criteria | Inclusion reason | Exclusion reason |
| --- | --- | --- |
| Population | - Adults (≥18 years); and - Primary hypercholesterolemia or mixed dyslipidaemia; and/or - Atherosclerotic cardiovascular disease (established disease or at high risk) | - Patients without cardiovascular disease - Patients who are not at high or very risk of cardiovascular risk - Paediatric population |
| Interventions | - Bempedoic acid - Bempedoic acid in fixed-dose combination with ezetimibe - Bempedoic acid in combination with any other lipid-lowering therapies (such as statins, ezetimibe, alirocumab, evolocumab, or inclisiran) | - Studies that do not investigate one of the interventions of interest |
| Comparators | - Any or no comparator | - Not applicable |
| Outcomes | - Change in LDL-C, hsCRP - Cardiovascular outcomes: individual or composite occurrences of the following:   - Myocardial infarction   - Angina: stable, unstable   - Cardiovascular death (including coronary heart disease death)   - Coronary revascularisation   - Stroke: fatal, nonfatal, haemorrhagic, ischaemic - All-cause Mortality/death - Treatment discontinuation - Any adverse event(s) | - Studies that do not report at least one of the outcomes of interest |
| Study design | - Prospective observational studies (including single arm studies) - Retrospective observational studies - Health insurance or claims analysis - Electronic medical record analysis - Chart review(s) - Registry studies - Pragmatic clinical trials - Cross-sectional studies - Patient or physician surveys - Case-series (>20 patients) | - Animal or in-vitro studies - Narrative reviews - Randomised controlled trials - Non-randomised clinical trials - Case reports - Commentaries and letters - Consensus reports |
| Timeframe | - Full-texts: no limit - Conference search: 2022 to 2024 | - Full-texts: none - Conference search: 2021 or earlier |
| Language | - English-language publications | - Non-English-language publications |

Supplementary Table 4. Newcastle–Ottawa Scale Risk of Bias Results

|  | Selection | | | | Comparability |  | Outcomes | |  |  |
| --- | --- | --- | --- | --- | --- | --- | --- | --- | --- | --- |
| Study Name | **1** | **2** | **3** | **4** | **5** | **6** | | **7** | **8** | **Total** |
| Russo 2025 | 1 | 0 | 0 | 0 | 1 | 1 | | 0 | 1 | 4 |
| Rana 2025 | 1 | 0 | 1 | 1 | 0 | 1 | | 1 | 1 | 6 |
| Ramachandran 2024 | 1 | 0 | 1 | 1 | 0 | 1 | | 1 | 1 | 6 |
| Makhmudova 2023 | 1 | 0 | 0 | 1 | 0 | 1 | | 1 | 1 | 5 |
| Warden 2022 | 1 | 0 | 1 | 1 | 0 | 1 | | 1 | 1 | 6 |
| Mahajan 2024 | 1 | 0 | 1 | 1 | 0 | 1 | | 1 | 1 | 6 |
| Marazzi 2024 | 1 | 0 | 1 | 1 | 0 | 1 | | 1 | 0 | 5 |
| Jakubowska 2024 | 1 | 0 | 1 | 1 | 0 | 1 | | 1 | 1 | 6 |

Orange = moderate quality; Red = low quality

1 – Representativeness of the exposed cohort
2 – Selection of the non-exposed cohort
3 – Ascertainment of exposure
4 – Demonstration that outcome of interest was not present at start of study
5 – Comparability of cohorts on the basis of the design or analysis
6 – Assessment of outcome
7 – Was follow-up long enough for outcomes to occur
8 – Adequacy of follow-up of cohorts
